# Application of particle swarm optimization in optimal placement of tsunami sensors

**DOI:** 10.7717/peerj-cs.333

**Published:** 2020-12-18

**Authors:** Angelie Ferrolino, Renier Mendoza, Ikha Magdalena, Jose Ernie Lope

**Affiliations:** 1 Institute of Mathematics, University of the Philippines Diliman, Quezon City, Philippines; 2 Faculty of Mathematics and Natural Sciences, Institut Teknologi Bandung, Bandung, Indonesia

**Keywords:** Particle swarm optimization, Nonlinear shallow water equations, Tsunami sensors, Tsunami early warning system, Heuristic algorithm, Finite volume method

## Abstract

Rapid detection and early warning systems demonstrate crucial significance in tsunami risk reduction measures. So far, several tsunami observation networks have been deployed in tsunamigenic regions to issue effective local response. However, guidance on where to station these sensors are limited. In this article, we address the problem of determining the placement of tsunami sensors with the least possible tsunami detection time. We use the solutions of the 2D nonlinear shallow water equations to compute the wave travel time. The optimization problem is solved by implementing the particle swarm optimization algorithm. We apply our model to a simple test problem with varying depths. We also use our proposed method to determine the placement of sensors for early tsunami detection in Cotabato Trench, Philippines.

## Introduction

While not the most prevalent among all natural disasters, tsunamis rank higher in scale compared to any others because of its destructive potential. Tsunamis are a series of ocean waves prompted by the displacement of a large volume of water. They can be generated by earthquakes, landslides, volcanic eruptions and even meteor impacts, although they mostly take place in subduction zones caused by underwater earthquakes. The sudden motion created by the said disturbances gives an enormous shove to the overlying water, which then travels away from the source.

Tsunamis usually have small initial amplitudes, so they can go unnoticed by sailors. However, they have far longer wavelengths unlike normal sea waves. Since the rate at which a wave loses its energy is inversely proportional to its wavelength, tsunami lose little energy as they propagate. Therefore, out in the depths of the ocean, tsunamis can travel at high speeds and cross great distances with only little energy loss (Liu, 2009). As they approach shallower waters, they slow down, and begin to grow in height. Once they crash ashore, they can cause widespread property damage, destruction of natural resources, and human injury or death.

Given the devastating potential of tsunamis, it is of great importance to develop techniques regarding risk assessment and damage mitigation. There are a number of methods focused on these in literature (Goda, Mori & Yasuda, 2019; Park et al., 2019; Goda & Risi, 2017). In this research, our goal is to determine the optimal locations of sensors for the earliest detection of tsunami waves. Through this, we can enable early and effective response to reduce casualties.

Traditionally, tide gauges, typically placed in a pier, are used for tsunami detection. They are employed to determine trends in the mean sea level, tidal computation, and harbor operations and navigation. However, since they are located in areas where tsunamis still have very high energy, they are frequently destroyed and fail to report the incoming tsunami and its characteristics. Furthermore, as they are near the coast, tsunami confirmation may arrive too late for timely evacuation measures which may lead to an ineffective local response (Barrick, 1979). Hence, we instead consider seafloor-mounted sensors, particularly the bottom pressure recorders (BPRs). These sensors are capable of detecting tsunamis, even in open oceans, by measuring variations in hydrostatic bottom pressure. They use an acoustic coupling to transmit data from the seafloor to the surface buoys, which then relay via satellite the information to a land-based station (Eble & Gonzalez, 1990). Further details on these tsunami sensor networks can be found in Rabinovich & Eblé (2015) and Nagai et al. (2007).

Studies on where to optimally place sensors of tsunami observation networks are limited. Some considered various technical aspects supported by expert judgments such as financial limitations (Araki et al., 2008) and legal aspects on geographical boundaries (Abe & Iwamura, 2013). Attempts to maximize the accuracy of prediction of some tsunami parameters (e.g., source, amplitude, etc.) were proposed in Mulia, Gusman & Satake (2017), Meza, Catalán & Tsushima (2020), Mulia et al. (2017), Saunders & Haase (2018). There are also researches that incorporated the effectiveness of tsunami warnings. In Omira et al. (2009), Schindelé, Loevenbruck & Hébert (2008), possible locations of sensors while considering installation constraints were investigated. However, the researchers did not apply optimization algorithms to solve the problem. In Braddock & Carmody (2001) and Groen, Botten & Blazek (2010), the optimal location of sensors among the candidate detection sites were proposed. In their investigations, they did not take into account bathymetric data since a fixed wave travel speed was used.

Astrakova et al. (2009) developed a problem of placing sensors optimally to detect tsunami waves as early as possible. However, the algorithm of computation for the wave travel time (from a source point to a point of interest) is based on the Huygen’s principle, which is computationally expensive. In Ferrolino, Lope & Mendoza (2020), wave velocity approximation was based on wave front kinematics. In this article, we compute for the wave travel time using the 2D nonlinear shallow water equations (SWE). This approach allows us to more accurately compute the tsunami detection time.

The 2D nonlinear SWE is generally used in the numerical simulation of tsunami propagation from the open ocean to the coast (Ulutas, 2012). They are accurate for solving long wave propagation (i.e., waves whose vertical length scale is much greater than their horizontal length scale), and run-up and inundation problems due to the introduction of nonlinear terms, which are essential in tsunami modeling. Moreover, they can take into account many types of tsunami generation (Zergani, Aziz & Viswanathan, 2015; LeVeque, George & Berger, 2011). In solving the 2D nonlinear SWE, we use the finite volume method in a staggered grid, with a momentum conservative scheme, as introduced in Pudjaprasetya & Magdalena (2014), Stelling & Duinmeijer (2003), Magdalena, Rif’atin & Reeve (2020), Magdalena, Iryanto & Reeve (2018), Andadari & Magdalena (2019), Magdalena, Pudjaprasetya & Wiryanto (2014), and Magdalena & Rif’atin (2020). This method is very effective and robust because it is well-balanced, conservative, and capable of handling complex bathymetry and topography.

We apply the Particle Swarm Optimization (PSO) in solving the optimal sensor location problem. This is a population-based algorithm inspired from the intelligent collective behavior of animal groups such as flocks of birds. Studies show that these animals are capable of sharing information among their group, and such ability grants them a survival advantage (Kennedy & Eberhart, 1995; Shi & Eberhart, 1998). PSO has gained much attention in solving optimization problems because it is easy to implement, computationally inexpensive, robust, and quick in convergence.

We first test our proposed approach on benchmark problems before using it to determine the optimal placement of sensors in the Cotabato trench, located in Mindanao, Philippines. The Philippines is vulnerable to tsunamis due to the presence of offshore faults and trenches (Valenzuela et al., 2020). To date, a total of 41 tidal waves classified as tsunamis have struck the country since 1589 (Bautista et al., 2012). Moreover, compared to neighboring countries, it has received less attention in tsunami research despite having regions with high seismic activity (Løvholt et al., 2012).

The remainder of this article is structured as follows: the methods implemented to solve the problem of optimal sensor placement are discussed in the “Methodology”. “Numerical Results” obtained on different domains are then presented. Finally, we conclude our work and provide suggestions for future research.

## Methodology

### The tsunami sensor optimal placement problem

**Figure 1:**
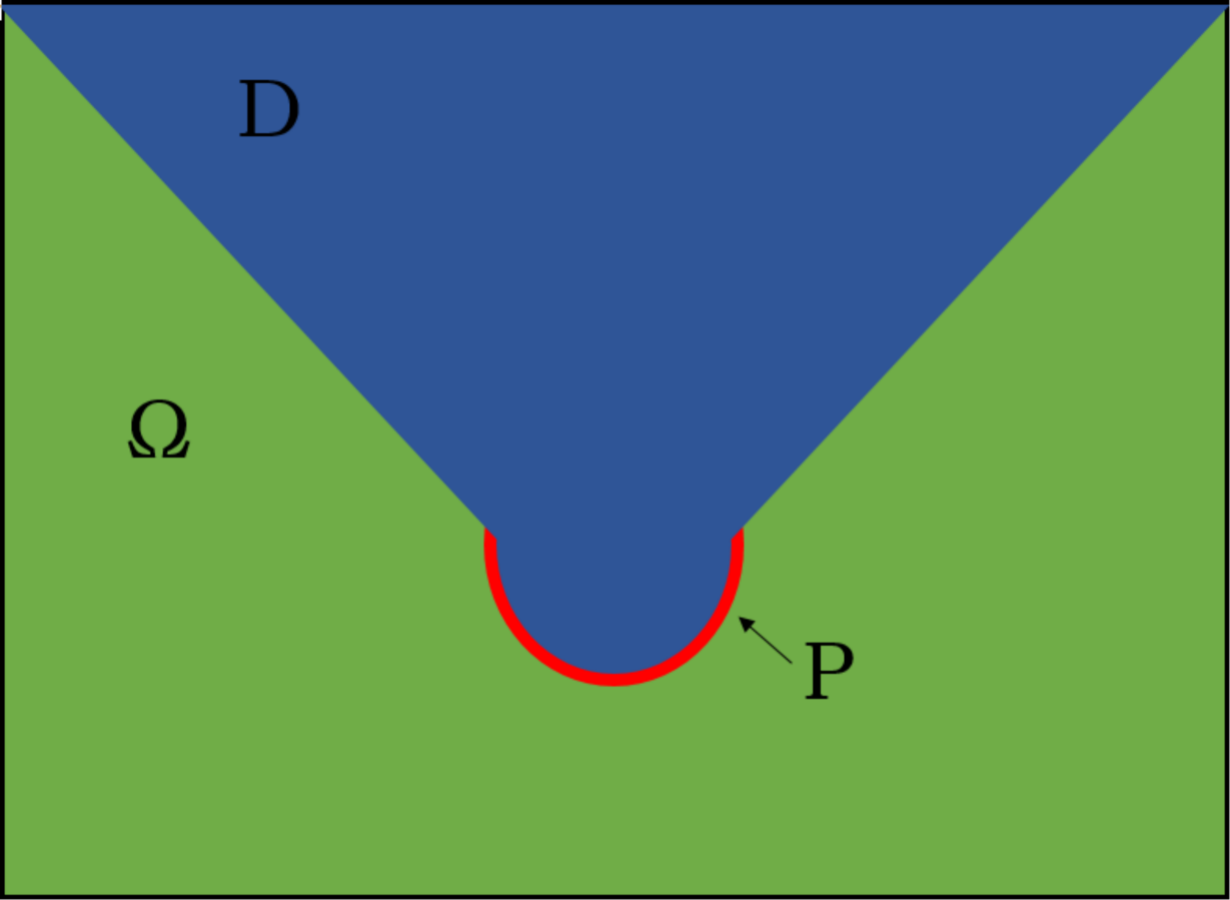
Illustration of a domain W with parts of water D (blue) and subduction zone P (red).

Consider the domain with water areas Ω, and the subduction zone **P**. Let **D** be the part of the domain Ω where the sensors can be stationed. A sample illustration is shown in Fig. 1. Our goal is to determine the optimal placement of *L* sensors for the earliest detection of tsunami waves, arising from any source point in **P**. We denote {*p*_*j*_}^*P*^_*j*__= 1_ as the points located in the subduction zone *P*, where *p*_*j*_ = (*x*_*j*_*,y*_*j*_). Moreover, let {*q*_*i*_}^*L*^_*i* =__1_ denote the sensors to be placed in **D**, where *q*_*i*_ = (*x*_*i*_*,y*_*i*_). We also let the configuration *Q* = {*q*_*1*_*,…,q*_*L*_} of *L* sensors represent a potential solution to the problem of interest.

Assume a source point *p*_*j*_ ∈ **P** generates some perturbation. The wave caused by such event will move away from the source, arriving at some water point *x* ∈ **D**. We denote

(1) $${\tau} ({p_j},x): = {\rm travel\; time\; from }\;{p_{j\;}}{\rm to }\;x.$$

To calculate the travel time τ in (1) from *p*_*j*_ to an arbitrary point *x* ∈ **D**, we solve the 2D nonlinear SWE by initiating the wave from *p*_*j*_. The time it takes for the wave to reach *x* will be τ*(p*_*j*_*, x)*. The numerical solution of the 2D nonlinear SWE is discussed in the next section. The time it takes for **Q** to detect a tsunami from *p*_*j*_ is computed as

$$t({p_j},{\bf Q}) = \mathop {\min }\limits_{1 \le i \le L} \;\tau ({p_j},{q_i}).$$

Here, we take the minimum travel time over all *L* sensors since we want detection by one sensor to be as early as possible. Next, we need to take into account all possible source points *p*_*j*_ to obtain the tsunami registration time *T*(**Q**) by configuration **Q**, that is, we set

$$T({\bf Q}) = \mathop {\max }\limits_{1 \le j \le P} \;t({p_j},{\bf Q}),$$

where the maximum of the earlier expression is taken over all source points *p*_*j*_. Thus, we formulate the optimization problem as follows:

Given *L* sensors, find Q* *= {q*_*1*_*,…,q*_*L*_*}* that minimizes *T*(**Q**) over **D**, or equivalently,

(2) $${{\bf Q}^{\rm \ast }} = {\mathop {\rm arg\,min}\limits_{{\bf Q} \in {\bf D}}}T({\bf Q}).$$

The minimization problem above is based on the formulation in Astrakova et al. (2009).

### The shallow water equations

Shallow water equations are a set of hyperbolic partial differential equations that describe fluid flow (Sadaka, 2012). They are often used to describe geophysical flows (such as rivers, lakes, coastal areas, etc.), rainfall runoff, tsunami propagation, or even atmospheric flows, given the appropriate initial conditions and source terms (Backhaus, 1983; Cea & Bladé, 2015; Rahman & Mandokhail, 2018; Galewsky, Scott & Polvani, 2004; Fu et al., 2017; Liu et al., 2008, 1995). SWE are derived from the Navier–Stokes equations, with the main assumption that the horizontal length scale is much greater than the vertical length scale. The 2D nonlinear shallow water equations are given by

$${h_t} + {(hu)_x} + {(hv)_y} = 0$$

$${(hu)_t} + {\left( {h{u^2} + \displaystyle{1 \over 2}g{h^2}} \right)_x} + {(huv)_y} = gh{z_x}$$

$${(hv)_t} + {(huv)_x} + {\left( {h{v^2} + \displaystyle{1 \over 2}g{h^2}} \right)_y} = gh{z_y}$$

Here, *h* is the local water depth, *u*,*v* are the velocities in the *x* and *y* directions, respectively, *g* = 9.81 m/s^2^ is the gravitational acceleration, and ∇*z* is the bed profile. An equivalent form of this system is given by

(3) $${h_t} + {(hu)_x} + {(hv)_y} = 0$$

(4) $${u_t} + u{u_x} + v{u_y} + g{{\rm \eta}_x} = 0$$

(5) $${v_t} + u{v_x} + v{v_y} + g{{\rm \eta}_y} = 0$$

derived by setting *h* = η + *z* where η is the surface elevation. Note that we can rewrite *uu*_*x*_, *vu*_*y*_, *uv*_*x*_, and *vv*_*y*_ in terms of *_u_q* = *hu*, *_v_q* = *hv*, *u*, *v*, and *h*, that is

(6) $$u{u_x} + v{u_y} = \displaystyle{1 \over h}(({{}_u}qu{)_x} - {{}_u}{q_x}u) + \displaystyle{1 \over h}(({{}_v}qu{)_y} - {{}_v}{q_y}u)$$

(7) $$u{v_x} + v{v_y} = \displaystyle{1 \over h}(({{}_u}qv{)_x} - {{}_u}{q_x}v) + \displaystyle{1 \over h}(({{}_v}qv{)_y} - {{}_v}{q_y}v).$$

A compilation of the analytical solutions of the shallow water equations (both 1D and 2D) for different cases are presented in Delestre et al. (2013). In this article, we solve the 2D nonlinear SWE numerically by implementing the finite volume method in a staggered grid with a momentum conservative scheme (Pudjaprasetya & Magdalena, 2014; Stelling & Duinmeijer, 2003), which will be discussed below. The solution of the SWE provides us the value of *h* at any mesh point in the domain in a given time. Hence, we can determine the time it takes for a disturbance to reach any mesh point in the domain for the first time (i.e., minimal time), which is the unknown in (1). We will use linear interpolation to determine wave travel time at any given point, using the three closest mesh points to this point.

**Figure 2:**
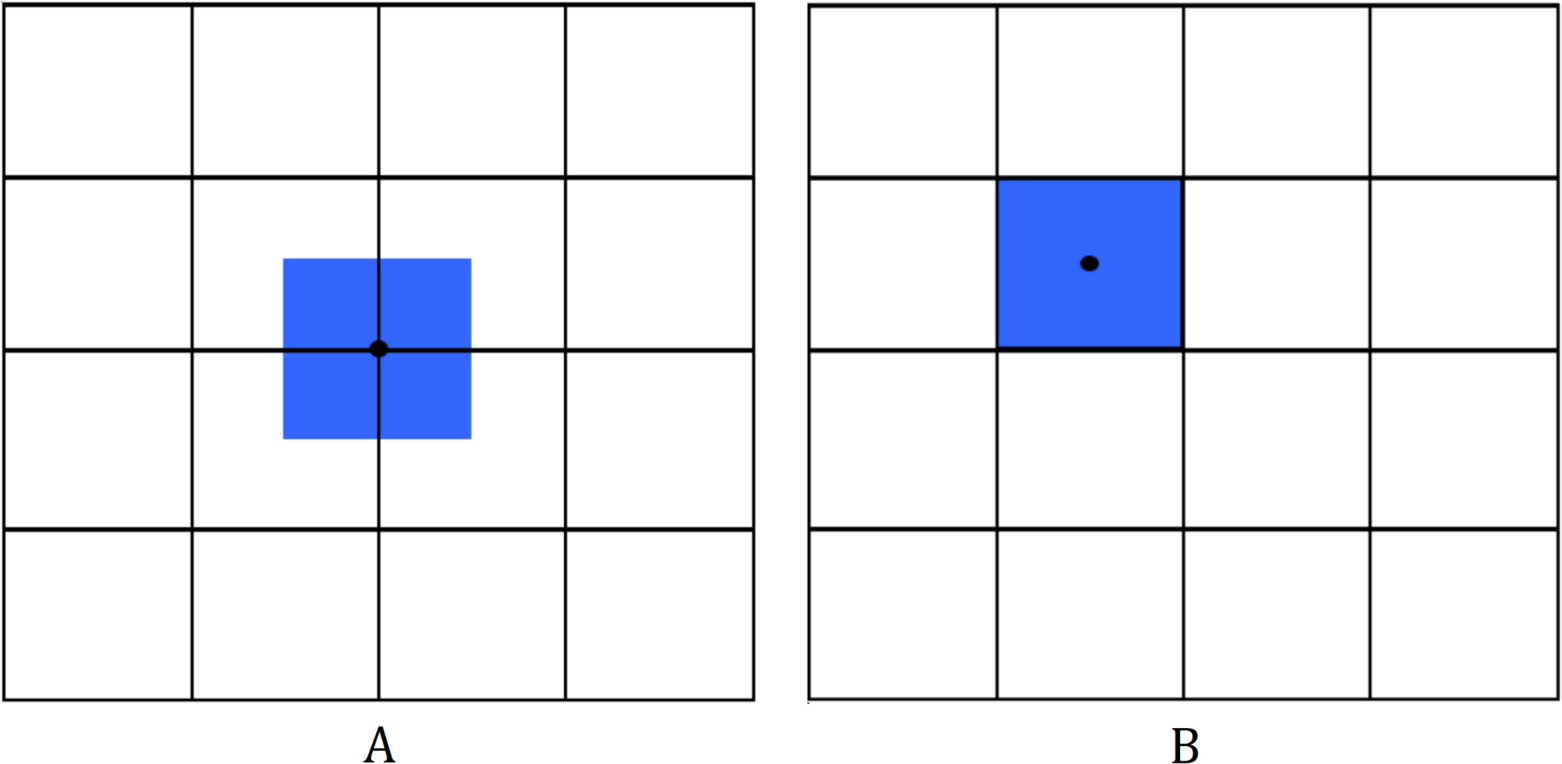
Mesh for the 2D finite volume method. Different schemes include (A) cell-vertex scheme; (B) cell-centered scheme.

The Finite Volume Method (FVM) is a method in solving partial differential equations (PDEs) by evaluating the conservative variables across the volume (LeVeque, 2002). The idea is to divide the domain into parts (see Fig. 2), which we will refer to as cells/control volumes, then integrate the equation over that volume. We then use Gauss’ Theorem to transform the volume integral into a surface integral. Evaluating this integral will give us the FVM discretization of a PDE.

Finite Volume Method has been extensively used in various fields such as heat and mass transfer, gas dynamics and fluid flow problems (Guedri et al., 2009, Lukáčová-Medvid’ová, Morton & Warnecke, 2002; Demirdžić & Perić, 1990). Its popularity as a discretization method stems from its high flexibility. The discretization is carried out directly in the physical domain without the need of transformation between the physical and computational system. Furthermore, it is easier to implement in unstructured meshes in comparison to other methods. Another important feature of FVM is that it preserves conservation, making it quite attractive when modeling problems where flux is of importance (Moukalled, Mangani & Darwish, 2016).

**Figure 3:**
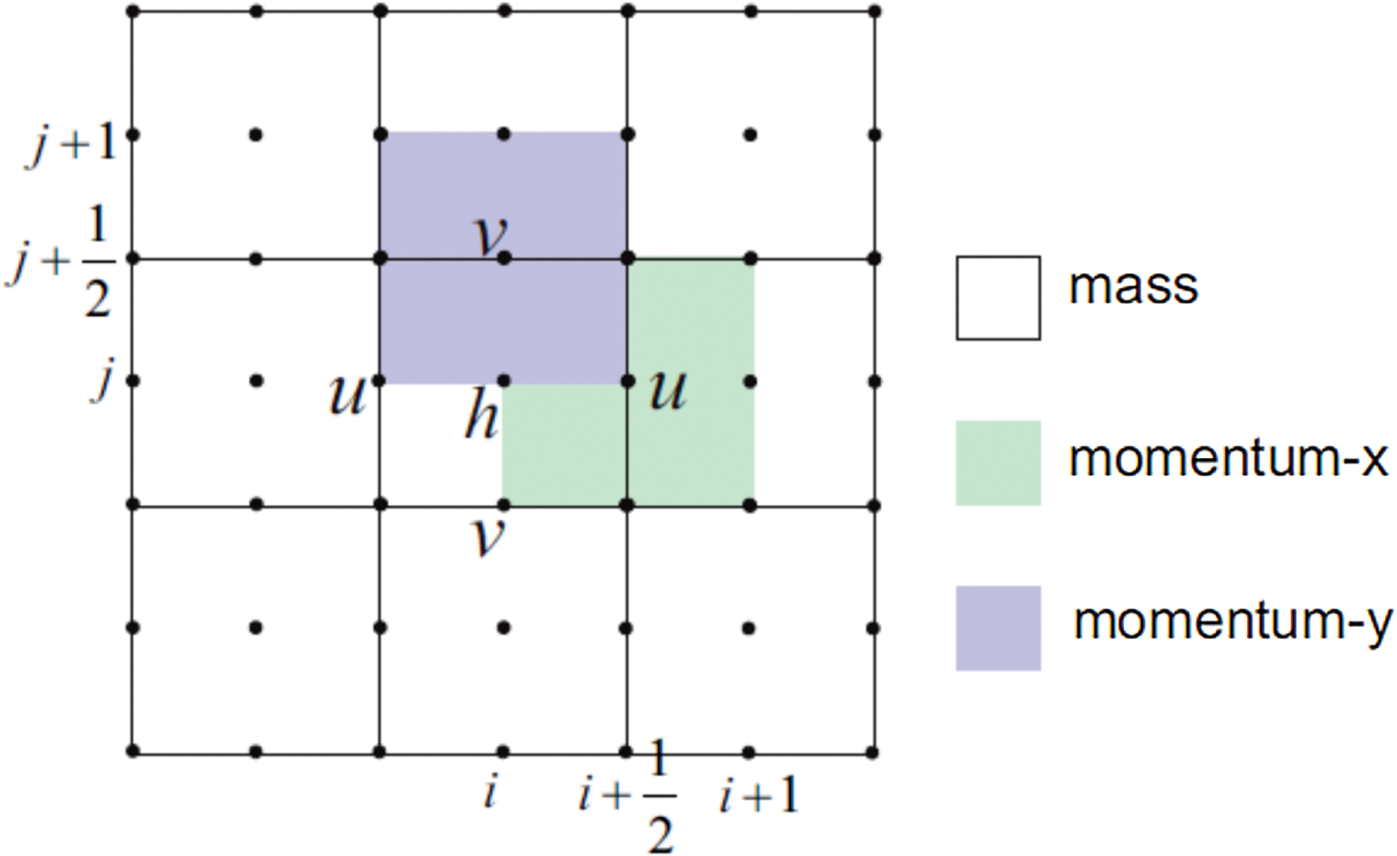
Illustration of the 2D staggered grid for the finite volume method.

We will use a staggered grid for the finite volume method. In a staggered grid, the scalar variables (mass, pressure, density, etc.) are defined at the cell centers, while the velocity or momentum variables are defined at the center of the cell faces. This is different from a collocated grid where all variables are stored in the same position (Harlow & Welch, 1965). Figure 3 shows the 2D staggered grid.

The implementation of FVM on a staggered grid on SWE is described as follows. Consider a rectangular spatial domain (0, *L*_*x*_) × (0, *L*_*y*_) with hard wall boundary conditions, that is, *u*(0, *y*, *t*) = *u*(*L*_*x*_, *y*, *t*) = 0 and *v*(*x*, 0, *t*) = *v*(*x*, *L*_*y*_, *t*) = 0. The domain is meshed with a grid of *M* × *N* cells. The center of each cell is denoted by *x*_*i*,*j*_, while the center of the bottom edge, top edge, left edge, and right edge of each cell are denoted by ${x_{i,j - \textstyle{1 \over 2}}}$, ${x_{i,j + \textstyle{1 \over 2}}}$, ${x_{i - \textstyle{1 \over 2},j}}$, and ${x_{i + \textstyle{1 \over 2},j}}$, respectively. Note that the sizes of *h*, *u*, and *v* are *M* × *N*, *M* × (*N* + 1), and (*M* + 1) × *N*, respectively.

The approximation of the mass Eq. (3) at the cell centered at *x*_*i*,*j*_ is

$$\displaystyle{{d{h_{i,j}}} \over {dt}} + \displaystyle{{^*{h_{i + \textstyle{1 \over 2},j}}{u_{i + \textstyle{1 \over 2},j}} - {^*}{h_{i - \textstyle{1 \over 2},j}}{u_{i - \textstyle{1 \over 2},j}}} \over {\Delta x}} + \displaystyle{{^*{h_{i,j + \scriptstyle{1 \over 2}}}{v_{i,j + \textstyle{1 \over 2}}} - {^*}{h_{i,j - \textstyle{1 \over 2}}}{v_{i,j - \textstyle{1 \over 2}}}} \over {\Delta y}} = 0$$

with upwind approximations

$$^*{h_{i + \textstyle{1 \over 2},j}} = \left\{ {\matrix{ {{h_{i,j}},} \hfill & {\quad if\,{u_{i + \textstyle{1 \over 2},j}} \ge 0} \hfill \cr {{h_{i + 1,j}},} \hfill & {\quad if\,{u_{i + \textstyle{1 \over 2},j}} \lt  0,} \hfill \cr {} \hfill & {} \hfill \cr } } \right. \qquad ^*{h_{i,j + \textstyle{1 \over 2}}} = \left\{ {\matrix{ {{h_{i,j}},} \hfill & {\quad if\,{v_{i,j + \textstyle{1 \over 2}}} \ge 0} \hfill \cr {{h_{i,j + 1}},} \hfill & {\quad if\,{v_{i,j + \textstyle{1 \over 2}}} \lt 0.} \hfill \cr {} \hfill & {} \hfill \cr } } \right.$$

The approximation of the momentum Eq. (4) is implemented at the cell centered at ${x_{i + \textstyle{1 \over 2},j}}$, and (5) at the cell centered at ${x_{i,j + \textstyle{1 \over 2}}}$. We use the relations (6) and (7) for the momentum conservative approximation of the advection terms. For positive flow directions *u* > 0, *v* > 0, we have

$$\displaystyle{{d{u_{i + \textstyle{1 \over 2},j}}} \over {dt}} + \displaystyle{{{_u}{{\overline q }_{i,j}}} \over {{{\overline h }_{i + \textstyle{1 \over 2},j}}}}\left( {\displaystyle{{{u_{i + \textstyle{1 \over 2},j}} - {u_{i - \textstyle{1 \over 2},j}}} \over {\Delta x}}} \right) + \displaystyle{{{_v}{{\overline q }_{i,j - \textstyle{1 \over 2}}}} \over {{{\overline h }_{i + \textstyle{1 \over 2},j}}}}\left( {\displaystyle{{{u_{i + \textstyle{1 \over 2},j}} - {u_{i + \textstyle{1 \over 2},j - 1}}} \over {\Delta y}}} \right) + g\displaystyle{{{{\rm \eta} _{i + 1,j}} - {{\rm \eta}_{i,j}}} \over {\Delta x}} = 0$$

$$\displaystyle{{d{v_{i,j + \textstyle{1 \over 2}}}} \over {dt}} + \displaystyle{{{_u}{{\overline q }_{i - \textstyle{1 \over 2},j}}} \over {{{\overline h }_{i,j + \textstyle{1 \over 2}}}}}\left( {\displaystyle{{{v_{i,j + \textstyle{1 \over 2}}} - {v_{i - 1,j + \textstyle{1 \over 2}}}} \over {\Delta x}}} \right) + \displaystyle{{{_v}{{\overline q }_{i,j}}} \over {{{\overline h }_{i,j + \textstyle{1 \over 2}}}}}\left( {\displaystyle{{{v_{i,j + \textstyle{1 \over 2}}} - {v_{i,j - \textstyle{1 \over 2}}}} \over {\Delta y}}} \right) + g\displaystyle{{{{\rm \eta} _{i,j + 1}} - {{\rm \eta}_{i,j}}} \over {\Delta y}} = 0,$$

where

$${\overline h _{i + \textstyle{1 \over 2},j}} = \displaystyle{1 \over 2}\Big({h_{i + 1,j}} + {h_{i,j}}\Big), \qquad \qquad {\overline h _{i,j + \textstyle{1 \over 2}}} = \displaystyle{1 \over 2}\Big({h_{i,j + 1}} + {h_{i,j}}\Big),$$

$${_u}{\overline q _{i,j}} = \displaystyle{1 \over 2}\Big( {{_u}{{\overline q }_{i + \textstyle{1 \over 2},j}} + {_u}{{\overline q }_{i - \textstyle{1 \over 2},j}}} \Big), \qquad {_u}{\overline q _{i + \textstyle{1 \over 2},j}} = {^*}{h_{i + \textstyle{1 \over 2},j}}{u_{i + \textstyle{1 \over 2},j}},$$

$${_v}{\overline q _{i,j}} = \displaystyle{1 \over 2}\Big( {{_v}{{\overline q }_{i,j + \textstyle{1 \over 2}}} + {_v}{{\overline q }_{i,j - \textstyle{1 \over 2}}}} \Big), \qquad {_v}{\overline q _{i,j + \textstyle{1 \over 2}}} = {^*}{h_{i,j + \textstyle{1 \over 2}}}{v_{i,j + \textstyle{1 \over 2}}},$$

$${_v}{\overline q _{i,j - \textstyle{1 \over 2}}} = {\overline h _{i,j - \textstyle{1 \over 2}}}{\overline v _{i,j - \textstyle{1 \over 2}}}, \qquad {\overline v _{i,j - \textstyle{1 \over 2}}} = \displaystyle{1 \over 2}\Big( {{v_{i + 1,j - \textstyle{1 \over 2}}} + {v_{i - 1,j - \textstyle{1 \over 2}}}} \Big),$$

$${_u}{\overline q _{i - \textstyle{1 \over 2},j}} = {\overline h _{i - \textstyle{1 \over 2},j}}{\overline u _{i - \textstyle{1 \over 2},j}}, \qquad {\overline u _{i - \textstyle{1 \over 2},j}} = \displaystyle{1 \over 2}\Big( {{u_{i - \textstyle{1 \over 2},{j + 1}}} + {u_{i - \textstyle{1 \over 2},j - 1}}} \Big).$$

Explicit schemes require a careful selection of the time step to fulfill the stability of the equation. One of the observations of Courant, Friedrichs & Lewy (1967) is that in order for solutions of a difference equation to converge to the solution of a partial differential equation, the difference scheme should use all the information contained in the initial data that influence the solution. This condition is called the CFL condition. The CFL number (also known as the Courant number) is given by

$$c = \displaystyle{{\Delta t \; {\rm max}({\lambda _x},{\lambda _y})} \over {{\rm min}(\Delta x,\Delta y)}}$$

To satisfy the CFL condition, the grid speed, given by $\textstyle{{\Delta x} \over {\Delta t}}$ and $\textstyle{{\Delta y} \over {\Delta t}}$, must be at least as large as the propagation speed in *x* and *y*, given by λ_*x*_ and λ_*y*_, respectively (Toro, 1999; Lax, 2013), that is, 0 ≤ *c* ≤ 1 holds. Thus, for the stability of SWE using FVM scheme on a staggered grid (Gunawan, 2015), we define the time step as follows:

$$\Delta t = c{\kern 1pt} \displaystyle{{\min (\Delta x,\Delta y)} \over {\mathop {\max }\limits_{i,j} \Big( {\displaystyle{1 \over {2{h_{i,j}}}}\Big| {u{q_{i + \textstyle{1 \over 2},j}} + u{q_{i - \textstyle{1 \over 2},j}}} \Big| + \sqrt {g{h_{i,j}}} ,\;\displaystyle{1 \over {2{h_{i,j}}}}\Big| {v{q_{i,j + \textstyle{1 \over 2}}} + v{q_{i,j - \textstyle{1 \over 2}}}} \Big| + \sqrt {g{h_{i,j}}} } \Big)}}.$$

The above numerical scheme has been validated through several benchmark tests in 1D and 2D (e.g., dam breaks, transcritical flows, wave shoaling), where the obtained numerical results are in very good agreement with the analytical solutions (Pudjaprasetya & Magdalena, 2014; Magdalena, Iryanto & Reeve, 2018; Magdalena, Erwina & Pudjaprasetya, 2015; Magdalena & Pudjaprasetya, 2014; Magdalena, Pudjaprasetya & Wiryanto, 2014).

### The particle swarm optimization algorithm

It was illustrated in Ferrolino, Lope & Mendoza (2020) that (2) is neither convex nor differentiable. Thus, gradient-based and local search algorithms may not be suitable to solve the problem. This justifies the use of population-based algorithms in determining the optimal locations.

For this work, we explore the use of the PSO algorithm (Kennedy & Eberhart, 1995; Shi & Eberhart, 1998) to solve the optimization problem presented in (2). This algorithm has been used to solve several optimization problems because of its speed and efficacy. It has been successfully used to solve unconstrained and constrained problems in a variety of fields such as engineering, communications theory, and operations research (Zhang, Wang & Ji, 2015). Other applications are in solving problems in clustering analysis (Chen & Ye, 2004), scheduling (Yu, Yuan & Wang, 2007; Zhang et al., 2014), facility location (Hajipour & Pasandideh, 2012) and vehicle routing (Belmecheri et al., 2013).

Particle Swarm Optimization is a population-based algorithm having a group of individuals (known as particles) moving in a search space to find the best solutions. The new positions of the particles are determined by their velocities, which are updated based on their own experience and the experience of neighboring particles. Through this, PSO can balance exploitation and exploration (He & Wang, 2007).

Algorithm 1 summarizes the steps of PSO, with some alterations proposed in Mezura-Montes & Coello (2011); Pedersen (2010). We use the MATLAB built-in command particleswarm in our implementations.

**Algorithm 1:** Particle swarm optimization algorithm.

**Input:** *npop*: swarm size, *v*: initial particle velocities within the range [−*r*,*r*], *nvars*: number of variables
**Output:** best particle location
1: Initialization. Create particles at random of size *npop* within bounds. Also, initialize inertia *ω* and *N* to *nbor* = max(2,*npop*×*frac*), where *nbor* is the minimum neighborhood size and *frac* is the minimum neighbors fraction. Set the stall counter *sc* = 0.
2: Evaluate the cost function *f* for all particles. Set *best* = min (*f*(*p*_*i*_)) where *p*_*i*_ is the position of particle *i*. In the latter iterations, *p*_*i*_ will be the position of the best objective function that particle *i* has found. Let *loc* be the location such that *best* = *f*(*loc*).
3: For each particle *i* in the swarm at position *x*_*i*_, do the following:
Select a subset *S* of *N* particles at random. Determine the best cost function value *cfbest*(*S*), and the position *xbest*(*S*) of *cfbest*(*S*).
Update the particle’s velocity to *v* = *ω v* + *w*_1_*u*_1_(*p* − *x*) + *w*_2_*u*_2_(*g* − *x*), where *v* is the previous velocity, *u*_1_,*u*_2_ are uniformly (0,1) distributed random vectors of size *nvars*, and *w*_1_,*w*_2_ are the weights for exploitation and exploration, respectively.
Update the particle’s position to *x* = *x* + *v* (implement thee bounds if necessary).
Calculate the cost function *F* = *f*(*x*). Set *p* = *x* if *F* < *f*(*p*).
4: Let *F* = min *F*(*k*) out of all *k* particles in the swarm. If *F* < *best*, set *best* = *F* and *loc* = *x*.
5: If the best cost function value is lowered, set *val* = 0. Otherwise, *val* = 1.
6: Update *N*. If *val* = 0:
Set *sc* = max(0, *sc* − 1).
Set *N* to *nbor*.
If *sc* < 2, set *ω* = 2 *ω*. If *sc* > 5, set *ω* = *ω*/2.
If *val* = 1:
Set *sc* = *sc* + 1.
Set *N* = min(*N* + *nbor*, *npop*).
7: Terminate if the stopping criterion has been satisfied. Else, go back to 3.

## Numerical Results

We now present the results of our numerical simulations. The proposed method was first tested on a rectangular domain with a semicircular subduction zone, and then later applied to a real-world problem. Because PSO is heuristic, ten independent runs were implemented for each scenario that was considered, and we present here the average values of the results of these runs. Moreover, we set the population to 50 * *L*, where *L* is the number of sensors, and the number of function evaluations to 10,000.

### Semicircle subduction zone with flat bottom

**Figure 4:**
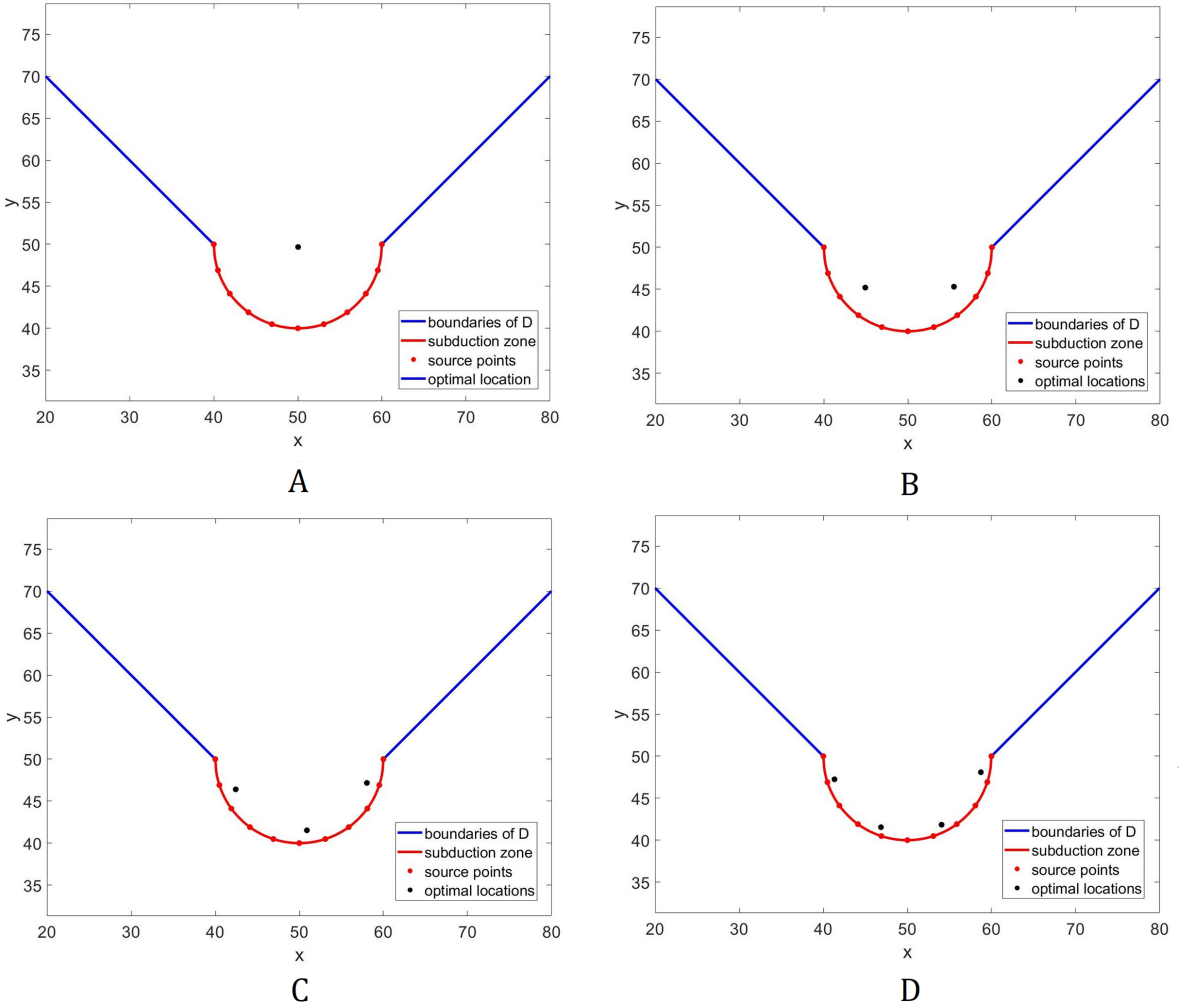
Optimal placement of sensors for the domain in Fig. 1. The bottom topography is assumed to be constant. Different numbers (*L*) of sensors are presented: (A) *L* = 1; (B) *L* = 2; (C) *L* = 3; (D) *L* = 4.

Consider the same domain shown in Fig. 1. Here, *D* is the water surface with constant depth and the rest is dry land. The optimal location of *L* = 1, …, 4 sensors are shown in Fig. 4. Note that for the case when *L* = 1, the optimal location of the sensor converged to the center of the circle. Moreover, as we employ more sensors, they tend to move closer to the subduction zone, and mimic its shape.

**Figure 5:**
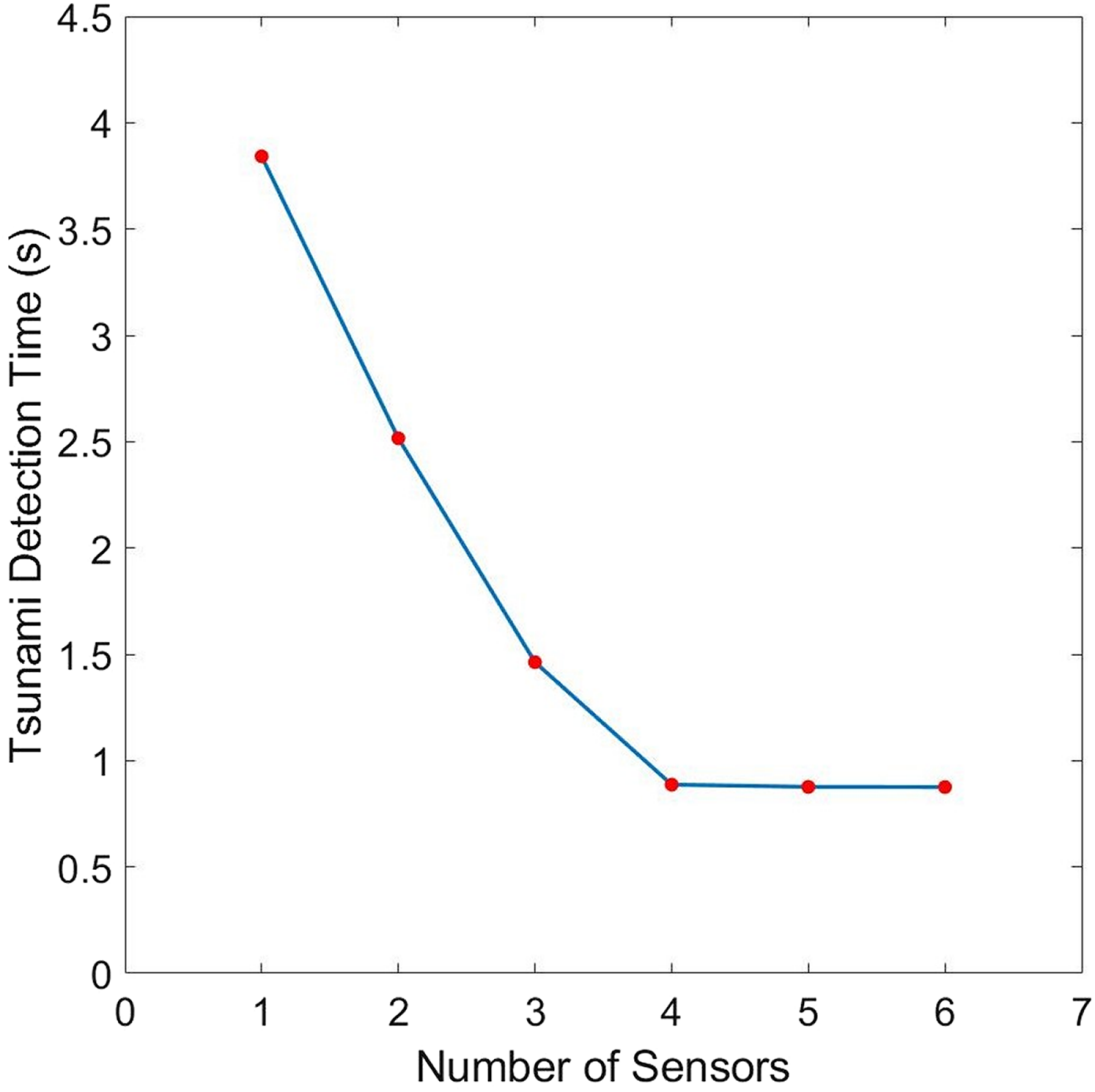
Comparison of the tsunami detection time for different number of sensors for the domain shown in Fig. 1. The bottom topography is assumed to be constant.

Now, we study how tsunami detection time will be influenced by changes in the number of sensors. Figure 5 plots the relationship between these two variables. We can see that detection time decreases as the number of sensors increases. Moreover, we can see from Table 1 that when we allocate additional sensors from 4, there is no significant difference compared to other cases. Hence, *L* = 4 sensors may be adequate in giving us fast time registration, without introducing too much costs.

**Table 1:** Improvement in the detection time of tsunami waves due to the increase in number (*L*) of sensors for the domain shown in Fig. 1. The bottom topography is assumed to be constant.

*L*	Detection time (s)	Improvement in time due to an additional sensor
1	3.84183	–
2	2.51604	1.32579
3	1.46286	1.05318
4	0.88702	0.57584
5	0.87654	0.01048
6	0.87565	0.00089

### Semicircle subduction zone with inverse tangent bottom

**Figure 6:**
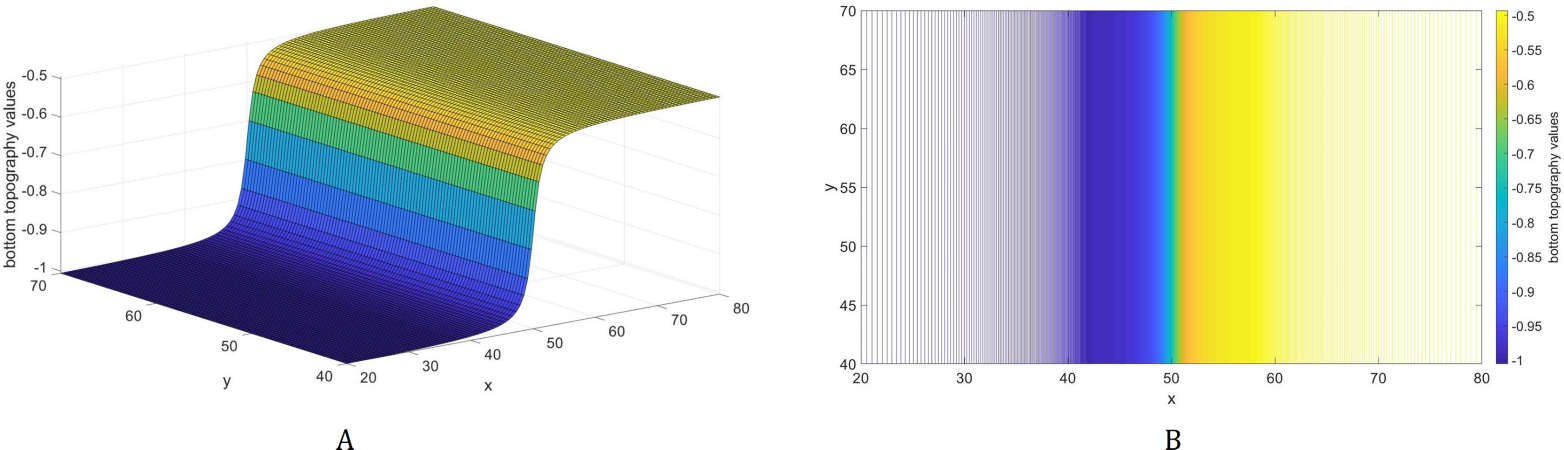
A bottom topography using the inverse tangent function. The bathymetric profile is shown in (A) while the contour plot is illustrated in (B).

**Figure 7:**
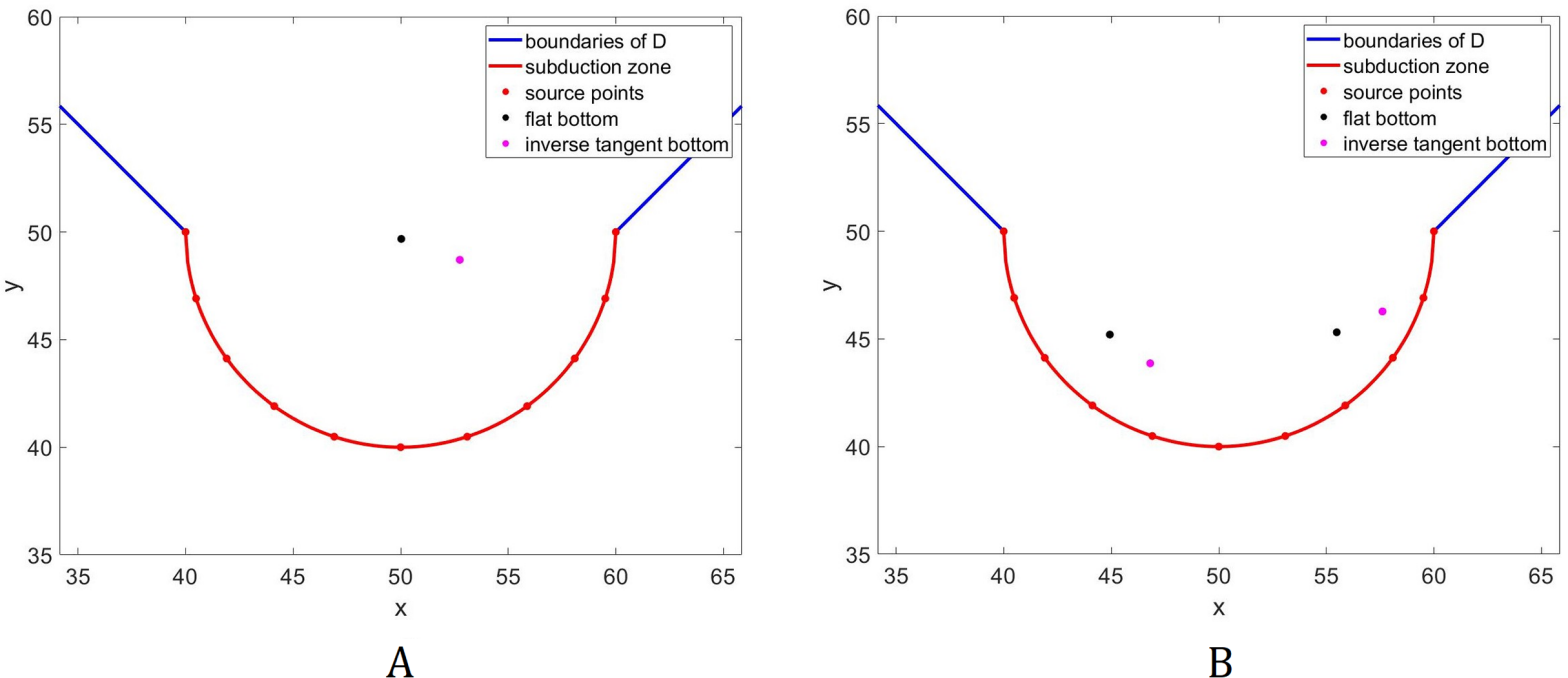
Comparison of the optimal location of *L* = 1 (A) and *L* = 2 (B) tsunami sensors using two different types of water depth: flat bottom topography (black) and inverse tangent bottom topography (magenta).

In this subsection, we examine how a different bathymetry will affect the optimal location of tsunami sensors. Specifically, we wish to investigate how the locations will change if there will be variations in the water depth. For this purpose, we consider an inverse tangent bottom topography as shown in Fig. 6. The optimal location of *L* = 1 and 2 sensors acquired using the inverse tangent bottom in comparison to the optimal locations attained using a flat bottom are presented in Fig. 7.

Observe in Fig. 7A that the sensor moves towards the shallower area. This phenomenon happens since waves travel slower in shallower areas, caused by the force exerted on them by the seabed. A similar trend can be observed for the two sensors as shown in Fig. 7B. Observe that the sensor in the right moves upwards and towards the shallower area. However, this will make tsunami detection time from the source points near the center longer. Thus, to accommodate these source points, the sensor on the left move rightwards and downwards. Note that this will not greatly affect tsunami detection time from the source points in deeper areas since waves travel much faster there.

### Optimal placement of sensors in Cotabato trench

The Cotabato trench is considered as one of the most dangerous major fault zones in the Philippines since it has high tsunamigenic potential. It is located near a southwestern coast of Mindanao, Philippines. Some examples of tsunamigenic earthquakes that occured in this trench are the 1918 Celebes Sea earthquake (M 8.0), the 1976 Moro Gulf earthquake (M 8.1), and the 2002 Mindanao earthquake (M 7.5) (Stewart & Cohn, 1979; Bautista et al., 2012).

**Figure 8:**
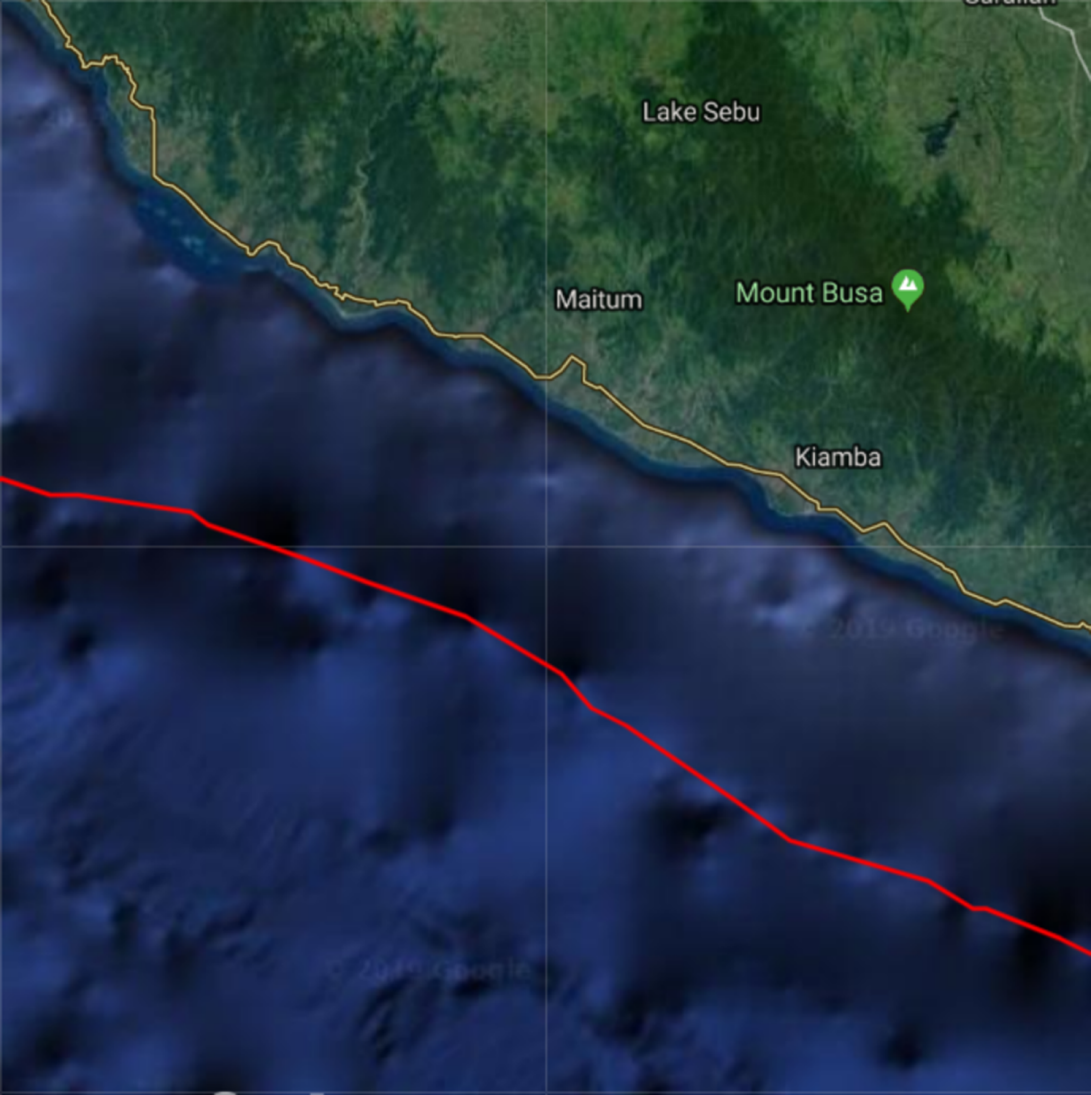
Top view of a portion of the Cotabato Trench. The red curve is the location of the subduction zone.

**Figure 9:**
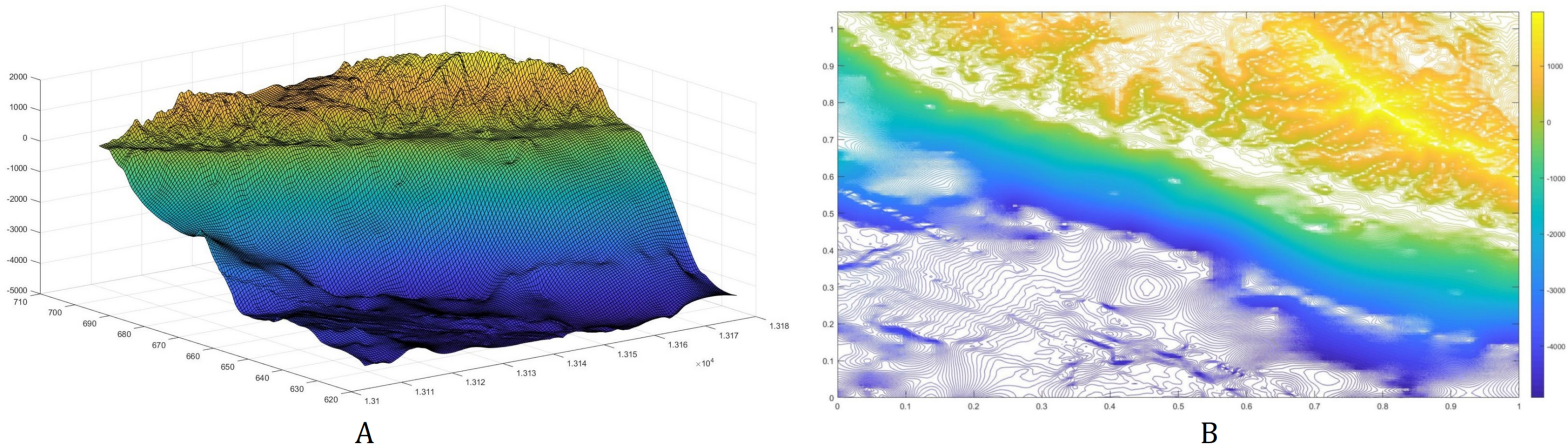
The bottom topography of Cotabato Trench. The bathymetric profile is shown in (A) while the contour plot is illustrated in (B).

Consider a portion of the Cotabato Trench as shown in Fig. 8. The top left corner has latitude 6.3142° and longitude 124.1083°, while the bottom right corner has latitude 5.6158° and longitude 124.8067°. The subduction zone **P** is illustrated by the red line, and **D** is the water surface above the subduction zone. The corresponding bathymetric profile of this trench is shown in Fig. 9. We obtained the bathymetric profile of Cotabato trench from the General Bathymetic Charts of the Oceans (GEBCO), https://www.gebco.net/.

**Figure 10:**
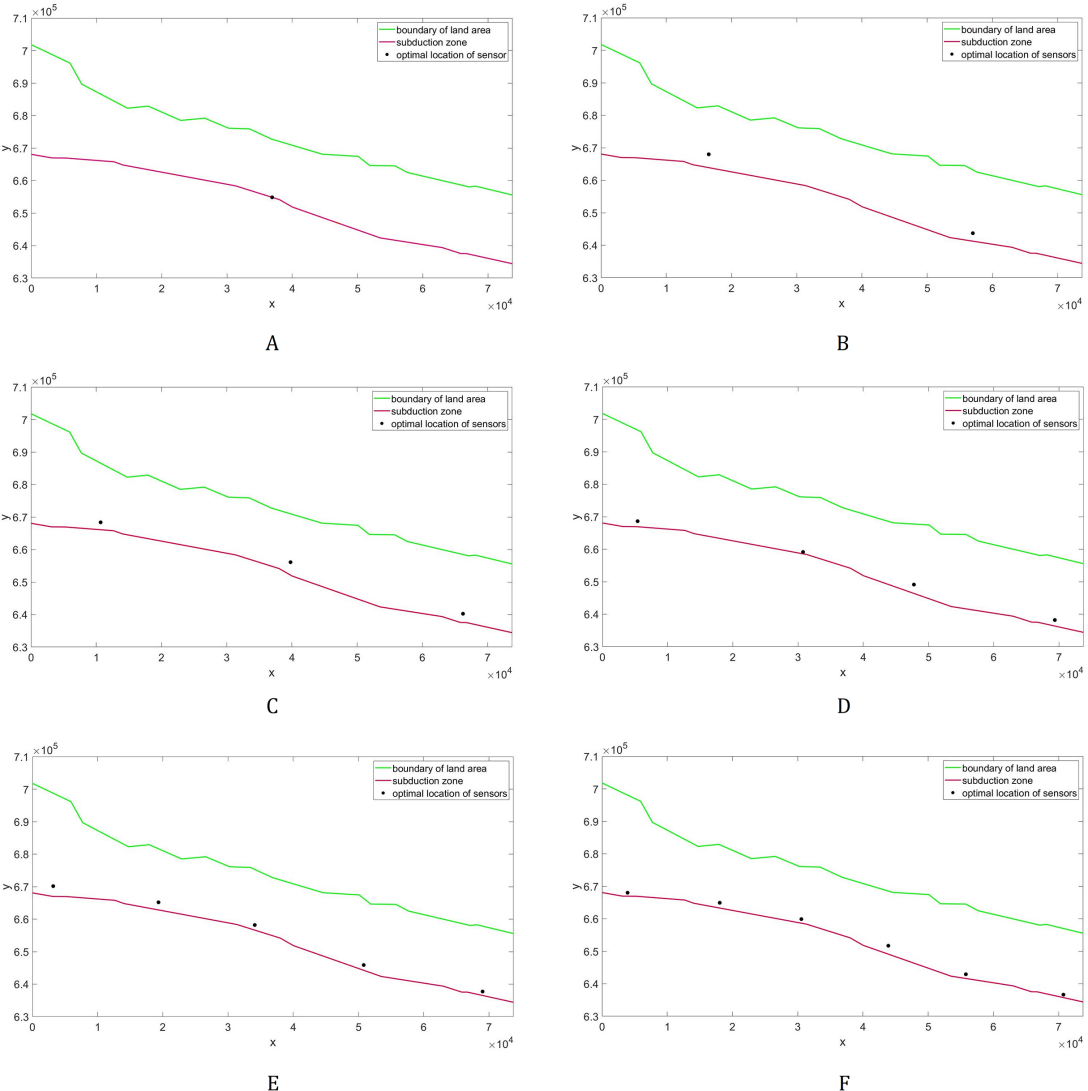
Optimal placement of the tsunami sensors in Cotabato Trench. Different numbers (*L*) of sensors are presented: (A) *L* = 1; (B) *L* = 2; (C) *L* = 3; (D) *L* = 4; (E) *L* = 5; (F) *L* = 6.

**Figure 11:**
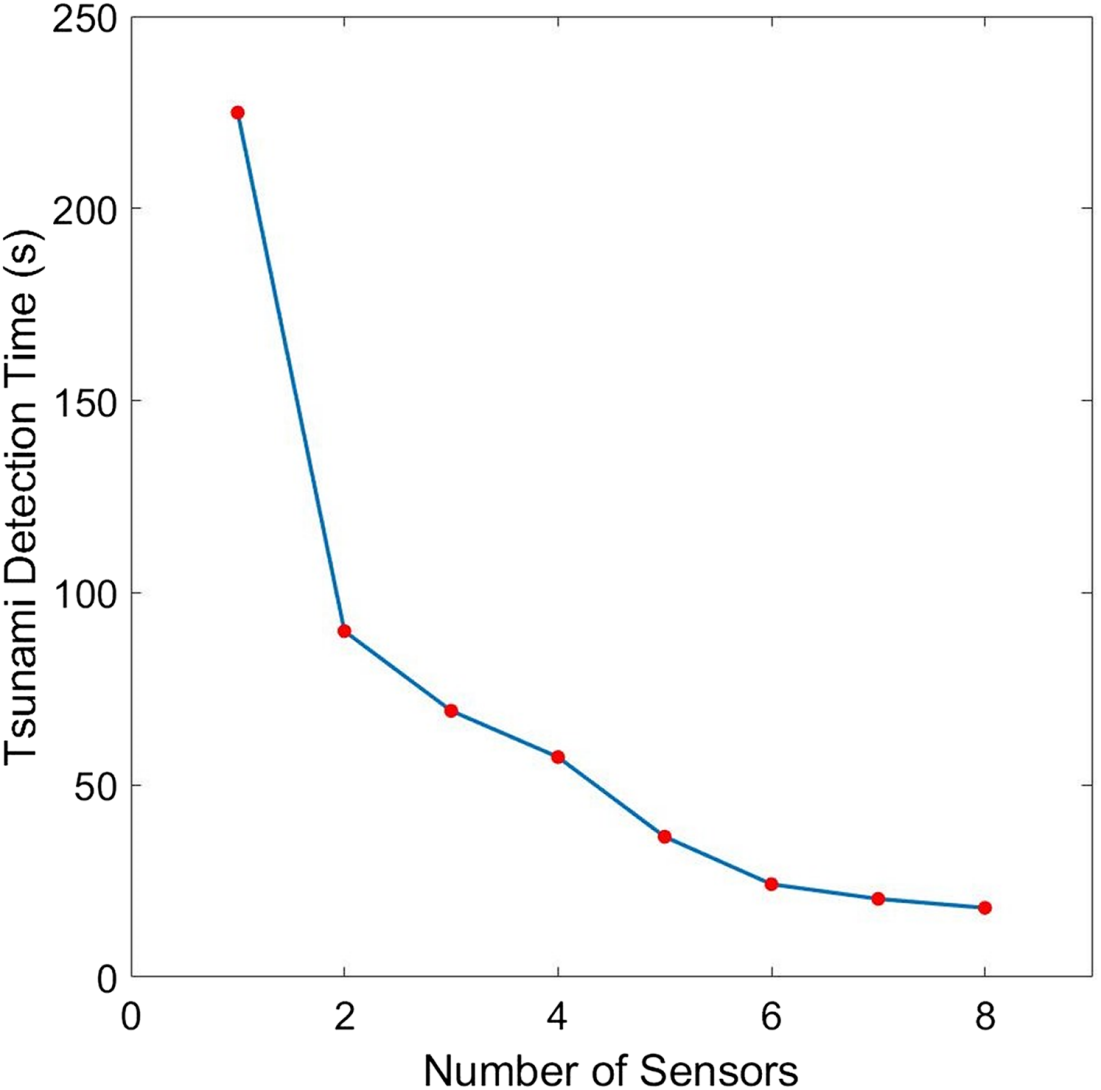
Comparison of the tsunami detection time for different number of sensors for the case when the domain is a portion of the Cotabato trench.

The placements of *L* = 1,…,6 sensor/s with minimum guaranteed time are illustrated in Fig. 10. Note here that as we employ more sensors, they become distributed along the subduction zone. The plot describing the relationship between detection time and the sensor’s number is shown in Fig. 11, while their corresponding values are presented in Table 2. Observe that increasing the number of sensors from 6 only shows little improvement in detection time. So, *L* = 6 sensors may be adequate in giving us fast time registration, without introducing additional costs.

**Table 2:** Improvement in tsunami detection time due to the increase in number (*L*) of sensors for the optimal placement of sensors in Cotabato trench.

L	Detection time (s)	Improvement in time due to an additional sensor
1	224.9557	–
2	90.0057	134.9500
3	69.2824	20.7233
4	57.2515	12.0309
5	36.5071	20.7444
6	24.1509	12.3562
7	20.3382	3.8127
8	18.0073	2.331

## Conclusion and Recommendations

The problem of placing sensors optimally is considered to provide the earliest detection of tsunami waves, which leads to effective warning and response by local communities. A more accurate calculation of wave travel time is proposed, which is done by solving the 2D nonlinear shallow water equations. Moreover, the PSO algorithm was implemented to solve the minimization problem. The proposed model was first tested to simple domains with varying bathymetries, comprising of a semicircle subduction zone. The calculated sensor locations for the benchmark problems are geometrically sensible. Afterwards, the model is applied to calculate the optimal placement of tsunami sensors in Cotabato Trench. The actual bathymetry profile of this trench is used to acquire a more realistic estimation of the wave travel time. We note here that our model can be used to any domain with any form of subduction zone or bathymetry. Moreover, it can consider other types of tsunami generation by changing the conditions in the 2D nonlinear shallow water equations.

Numerical results have shown an inverse relationship between the number of sensors and tsunami detection time. However, employing additional sensors implies more cost. Imposing constraints in cost and installation (e.g., depth, location) may be done in future works. Another recommendation is to consider maximizing tsunami warning efficacy and accuracy of estimation of tsunami parameters as additional objective functions.

## Supplemental Information

10.7717/peerj-cs.333/supp-1 Supplemental Information 1 MATLAB codes.

## Additional Information and Declarations

### Data Availability

The following information was supplied regarding data availability: We obtained bathymetric profile data from the Cotabato trench at the General Bathymetic Charts of the Oceans (GEBCO): https://download.gebco.net/. The top left corner has latitude 6.3142 degrees and longitude 124.1083 degrees, while the bottom right corner has latitude 5.6158 degrees and longitude 124.8067 degrees. We use the MATLAB built-in command particleswarm to solve the arising minimization problem. The details of the code can be found here: https://www.mathworks.com/help/gads/particleswarm.html
